# Insights From Google Play Store User Reviews for the Development of Weight Loss Apps: Mixed-Method Analysis

**DOI:** 10.2196/mhealth.8791

**Published:** 2017-12-22

**Authors:** Kerstin Frie, Jamie Hartmann-Boyce, Susan Jebb, Charlotte Albury, Rebecca Nourse, Paul Aveyard

**Affiliations:** ^1^ Nuffield Department of Primary Care Health Sciences Medical Sciences Division University of Oxford Oxford United Kingdom

**Keywords:** weight loss, mobile applications, telemedicine, consumer behavior

## Abstract

**Background:**

Significant weight loss takes several months to achieve, and behavioral support can enhance weight loss success. Weight loss apps could provide ongoing support and deliver innovative interventions, but to do so, developers must ensure user satisfaction.

**Objective:**

The aim of this study was to conduct a review of Google Play Store apps to explore what users like and dislike about weight loss and weight-tracking apps and to examine qualitative feedback through analysis of user reviews.

**Methods:**

The Google Play Store was searched and screened for weight loss apps using the search terms *weight loss* and weight track*, resulting in 179 mobile apps. A content analysis was conducted based on the Oxford Food and Activity Behaviors taxonomy. Correlational analyses were used to assess the association between complexity of mobile health (mHealth) apps and popularity indicators. The sample was then screened for popular apps that primarily focus on weight-tracking. For the resulting subset of 15 weight-tracking apps, 569 user reviews were sampled from the Google Play Store. Framework and thematic analysis of user reviews was conducted to assess which features users valued and how design influenced users’ responses.

**Results:**

The complexity (number of components) of weight loss apps was significantly positively correlated with the rating (*r*=.25; *P*=.001), number of reviews (*r*=.28; *P*<.001), and number of downloads (*r*=.48; *P*<.001) of the app. In contrast, in the qualitative analysis of weight-tracking apps, users expressed preference for simplicity and ease of use. In addition, we found that positive reinforcement through detailed feedback fostered users’ motivation for further weight loss. Smooth functioning and reliable data storage emerged as critical prerequisites for long-term app usage.

**Conclusions:**

Users of weight-tracking apps valued simplicity, whereas users of comprehensive weight loss apps appreciated availability of more features, indicating that complexity demands are specific to different target populations. The provision of feedback on progress can motivate users to continue their weight loss attempts. Users value seamless functioning and reliable data storage.

## Introduction

Mobile apps addressing health and fitness issues have emerged in large numbers over the last couple of years. In 2016, 231,000 mobile health (mHealth) apps were available on the Android and Apple app stores, of which nearly 100,000 had been added since 2015 [1]. The total number of worldwide downloads was estimated at 3.2 billion by the end of 2016 [1]. It is predicted that by 2020, over 2.5 billion people will have downloaded at least one health and fitness app onto their phone [1]. Many of these mHealth apps claim to provide weight loss support.

Given the wide reach of mHealth apps, researchers have started to explore their quality. In several app reviews, researchers have criticized the limited extent to which mHealth app features are grounded in evidence [2-7]. For instance, Pagoto and colleagues compiled 20 behavior change strategies from effective weight loss treatments and assessed how many of them were implemented by weight loss apps. They found that fewer than 20% of the strategies were used on average [8]. It is argued that the paucity of established behavioral techniques incorporated in the apps may limit their effectiveness in supporting users to change their behavior [9].

However, even the most evidence-based app is unlikely to be successful if it does not attract and retain users, as repeated and long-term interaction with an intervention is often critical for its effectiveness [10-12]. This is especially the case for self-monitoring interventions, where frequent self-monitoring is associated with greater weight loss [13-15]. Moreover, research shows that the popularity of an app is not necessarily associated with the extent of its evidence base [6]. Bardus and colleagues found that the popular mHealth apps on the app market were the ones that had good functionality and an appealing appearance, even though they were only of moderate quality content-wise [16]. Hence, for a health app to be engaging and effective, attention has to be paid to its presentation and design, not just its content. User retention is particularly challenging as the app store is a constantly changing market [17], meaning that users can easily switch apps. In fact, three-quarters of all downloaded health apps are used less than eleven times [18].

This paper explores which aspects of mHealth apps affect user satisfaction to provide guidance on how to keep users engaged. User reviews on app stores offer an opportunity to gain such insights from a large pool of people. Reviews often contain complaints, suggestions of change, and innovative ideas [19,20] and can therefore help identify the key aspects that affect user satisfaction. To the best of our knowledge, no academic analysis of user reviews on weight loss apps has yet been conducted. We aim to assess satisfaction with common features, explore user suggestions, and generate guidance for further weight loss app development. We focus on weight-tracking apps specifically, as the effectiveness of their main functionality, that is, self-monitoring, is dependent on the appeal and engagingness of the intervention [13-15]. Analyzing user reviews to improve likability and usability can therefore especially help improve the effectiveness of the respective apps. Moreover, self-monitoring is particularly likely to benefit from the technological advantages of smartphones, as they allow for instantaneous feedback, are easy and quick to use, are mobile and hence accessible most of the day, and their usage in social contexts is more acceptable than the usage of their nondigital alternatives [21-23]. Self-monitoring apps therefore provide an interesting example to study and advance developers’ capabilities in making use of the unique technological possibilities that mHealth apps provide to create appealing interventions.

## Methods

### Study Design

In a first step, weight loss apps were sampled from the Google Play Store. The components of these apps were coded to attain an overview of the most common weight loss app features. In a second step, the sample was screened for weight-tracking apps, which underwent a second and more detailed component coding. Finally, reviews were sampled from the weight-tracking apps and qualitatively analyzed according to users’ liking and disliking of components.

### Sampling Strategy—Weight Loss Apps

In March 2017, an electronic search on the Google Play Store was conducted using two search terms: *weight loss* and weight track*. The search was performed using an incognito browser in Google Chrome that was not connected to a Google account. Each search resulted in the display of 249 mobile apps. Due to the conceptual closeness of the search terms, the results partly overlapped.

For each results list, we sampled apps until no conceptually new apps were found. If saturation was not reached after the first 100 apps in the search results, more apps were considered from the list in batches of ten. This method resulted in the screening of 120 apps for the term *weight loss* and 110 apps for the term weight track*.

Where the same app was available in both a free and a paid version, we treated them as two separate apps. Properties of the mobile apps, such as app and developer name, app category, rating, number of reviews, number of downloads, version of the app, cost of the app, availability of in-app purchases, as well as the search term used and the position in each search results list were noted. The sampling process was conducted over 2 days, 1 day per search term.

### First Screening and Component Coding—Weight Loss Apps

Each mobile app was screened for the following three criteria: (1) whether it was targeted at people who want to lose or monitor their weight; (2) whether it had stand-alone functionality, meaning that it is usable without a membership subscription or ownership of specific devices; and (3) whether it was available in the English language. This screening resulted in the exclusion of 25 apps. Two more mobile apps were initially recorded but could no longer be found on the Google Play Store at the time of the screening process.

Details concerning the remaining 179 apps can be found in Multimedia Appendix 1. The mobile apps were manually coded by components using information available from the app description pages on the Google Play Store. We used an adapted version of the Oxford Food and Activity Behaviors (OxFAB) taxonomy (Table 1) that consists of 23 domains and can be used to classify self-help interventions for weight loss [24,25].

**Table 1:** Summary and definitions of components in the Oxford Food and Activity Behaviors (OxFAB) adaptation. The first column lists the adapted OxFAB components, the second column contains a definition for each adapted component and the third column contains the original OxFAB domains.

Adapted version		Definition	OxFAB domains^a^
Goal setting		Setting of a specific target, either behavioral or outcome-related; for example, weight loss goal	Goal setting
Feedback on goal progress		Feedback on the progress towards this target, quantifiable; for example, amount of weight left to lose to reach target	Non-existent
Impulse management		Components specifically designed to help users cope with impulses to eat or binge; for example, distraction	Impulse management: Acceptance
			Impulse management: Awareness of motives
			Impulse management: Distraction
Motivation		Components increasing motivation of users; for example, motivational quotes	Motivation
Planning content and scheduling of diet and activity		Components that provide support in planning and scheduling weight loss-related activities; for example, diet plans, physical activity challenges	Planning content
			Scheduling of diet and activity
**Behavioral strategies**			
	Physical activity	Advice on behavioral strategies related to physical activity that can help with weight loss; for example, raising knees higher during walking	Regulation: Allowances
			Regulation: Restrictions
			Regulation: Rule setting
	Dieting	Advice on behavioral strategies related to diet that can help with weight loss; for example, restricting portion sizes	Restraint
			Stimulus control
			Energy compensation
**Information**			
	Physical activity	Information on the benefit of a specific physical activity; for example, which muscle groups are trained with an exercise	Non-existent
	Dieting	Information on the nutritional value of foods; for example, amount of calories in a banana	Non-existent
**Prescriptive help**			
	Physical activity workout	Instructions for physical activity; for example, a workout	Non-existent
	Menus	Instructions related to food; for example, recipes, shopping lists	Non-existent
Calorie calculator		Component that calculates the daily energy expenditure or maximum amount of daily calories allowed	Non-existent
Reframing		App reframes weight loss as, for example, a change in lifestyle	Reframing
Reward		Reward for reaching a specific outcome, usually a gamification element; for example, winning badges, unlocking new components	Reward
**Self-monitoring**			
	Weight tracking	Component that logs weight measurements over time	Self-monitoring
	Body fat tracking	Component that logs body fat measurements over time	Self-monitoring
	Body measurements tracking	Component that logs body measurements over time	Self-monitoring
	Physical activity tracking	Component that logs physical activity over time	Self-monitoring
	Diet tracking	Component that logs diet over time	Self-monitoring
	Body fat calculator	Component that calculates body fat percentage	Self-monitoring
	BMI calculator	Component that calculates BMI	Self-monitoring
Reminder setting		Component that allows to set a reminder; for example, reminds to enter weight measurement	Non-existent
			
			
			
			
**Support**			
	Buddying	Social community component which allows users to link up with each other to pursue goals together; for example, compete with each other in a weight loss challenge	Support: Buddying
	Motivational	Social media linkage which allows users to share their progress with their friends and family	Support: Motivational
	Professional	Offers provision of direct support from a health professional; for example, possibility to chat to a dietician	Support: Professional
Weight management aids		Components that offer other weight management aids; for example, hypnosis, advice on weight loss body wraps	Weight management aids
Self-experimentation		App encourages users to try out different weight loss strategies; for example, experiment with different diets	Self-experimentation

^a^OxFAB domains “Imitation (modeling)“ and “Information seeking” were deleted from the adapted version.

In many cases, the mobile apps offered in-app purchases that enabled access to premium features. For this component coding, all premium features that were mentioned on the app description pages were considered.

The component coding was performed by two coders for 10.1% (18/179) of the mobile apps. Prevalence-adjusted and bias-adjusted kappa (PABAK) scores assessing the agreement between the coders showed fair to good results (mean PABAK=0.91). Disagreements between the coders were mostly based on differences in the conceptual understanding of the domain definitions. The definitions were subsequently clarified, which resulted in a slightly higher PABAK score (0.94). One coder performed the rest of the component coding.

### Second Screening and Component Coding—Weight-Tracking Apps

For the review analysis, the weight loss app sample was screened for weight-tracking apps. To be eligible, these apps (1) had to be primarily focused on weight-tracking (ie, have no other main function such as a detailed diet tracker or exercise instructor but could have additional minor features such as a body mass index [BMI] calculator and counter of glasses of water consumed), (2) had to have a user rating above 3 stars, and (3) had to have at least 1000 reviews. Of the 179 mobile apps that were coded by component, 15 met these criteria. We conducted another, more fine-grained coding of the components for the weight-tracking apps. Each app was downloaded from the Google Play Store to an HTC A9 smartphone (Android 7.0) and manually rated according to the existence of several features (Multimedia Appendix 2). No paid versions of the apps were purchased. Premium features were coded according to their mentioning in the app description pages on the Google Play Store or in the app itself.

### Review Sampling—Weight-Tracking Apps

**Figure 1:**
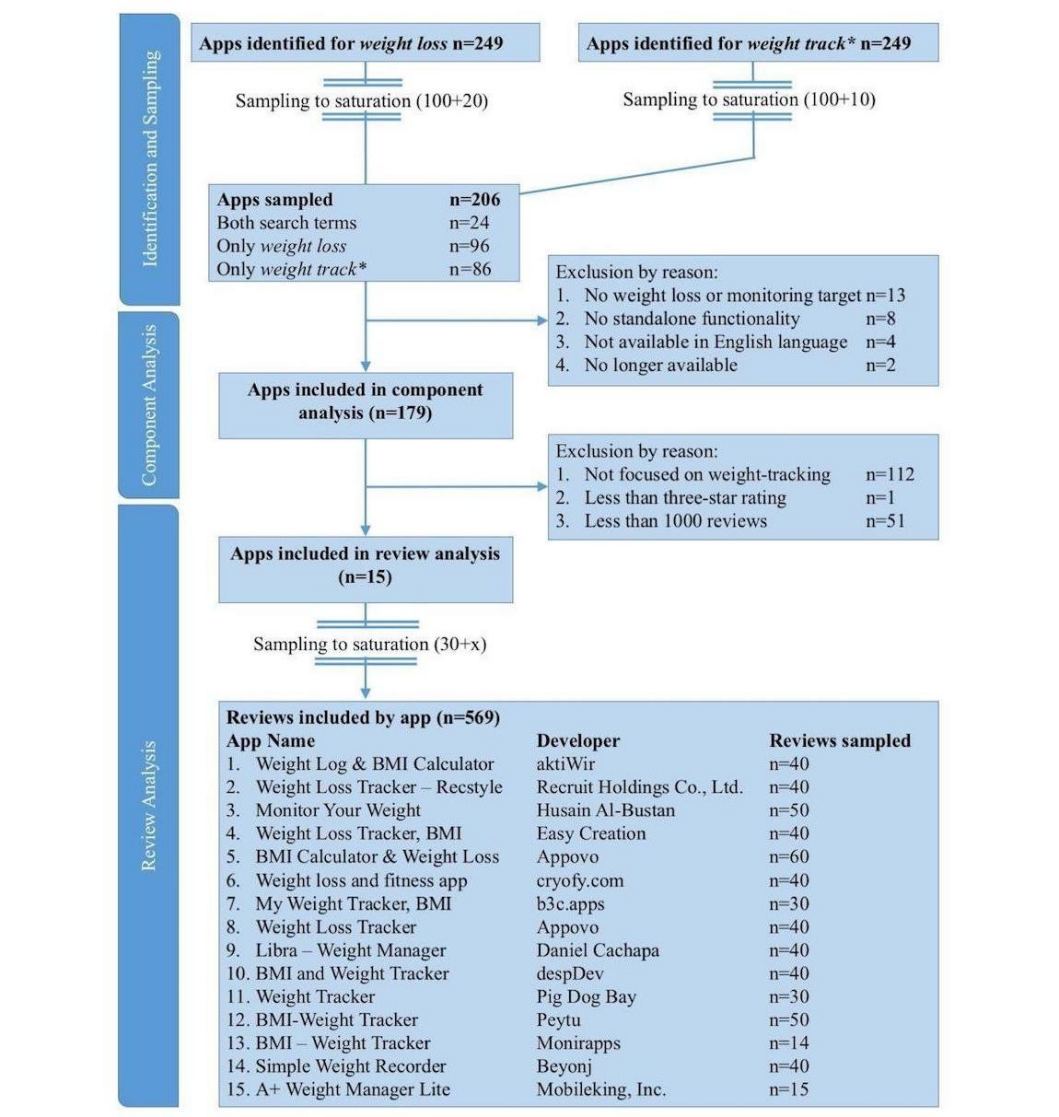
Screening and sampling procedure for this review.

In a next step, user reviews were sampled from the 15 mobile apps. Only reviews written in the English language were considered. A sampling to saturation strategy was employed, whereby we extracted the first 30 reviews displayed for each app when the reviews were sorted by helpfulness. If saturation was not reached after 30 reviews (ie, new aspects were still raised by the users in the last ten reviews), we continued extracting text from further reviews in batches of ten until no new information was gathered. For two of the 15 apps, less than 30 reviews included text, and thus, all available reviews with text were included in the analysis. Figure 1 displays the screening and sampling process for both the component coding and the review analysis.

### Data Analysis

Data was analyzed using a mixed-methods approach. Using data from the first component coding, Pearson correlations assessed the relationship between complexity of the app and popularity indicators (rating, number of reviews, and number of downloads). These calculations were performed using the Statistical Package for the Social Sciences (SPSS) version 24 (IBM Corp).

The reviews of weight-tracking apps were imported to and analyzed in NVivo (QSR International) using a framework and thematic analysis. With the framework analysis, our aim was to evaluate the popularity of the components present in weight-tracking apps. To this end, we set up a node structure, including main components from the second component coding. In the thematic analysis, further nodes, which were not covered by the framework analysis, were added according to emerging themes. Coding was performed by two independent coders for the first three apps, reaching high interrater reliability (κ=.83). The node structure was then collapsed, and the rest of the mobile apps were coded and analyzed by one researcher.

## Results

### Component Analysis Weight Loss Apps

#### Descriptive Analysis

In the first component analysis, we applied the adapted version of the OxFAB domains to 179 weight loss and weight management apps from Google Play Store. The apps covered on average 5.15 domains (standard deviation [SD] 3.33). More than half of the mobile apps had weight-tracking and goal setting functionalities. Other frequent domains were the BMI calculator and feedback concerning the progress toward a set goal (Table 2). Considering only the five tracking domains (ie, weight, body fat, body measurements, diet, and exercise), we found that the apps covered 1.21 domains on average (SD 1.29). Approximately one-third (n=62) of the apps focused on one tracking function only, another third incorporated two or more tracking components (n=52), and the last third did not have a tracking function at all (n=65).

#### Correlational Analysis

Using Pearson correlations, we found that the count of components was strongly positively correlated with rating (*r*=.25; *P*=.001), number of reviews (*r*=.28; *P*<.001), and number of downloads (*r*=.48; *P*<.001). Hence, the mobile apps that incorporated more components (ie, that were more complex) were more popular in our sample (see Table 3).

**Table 2:** Frequency with which the adapted Oxford Food and Activity Behaviors (OxFAB) components were coded in weight loss apps.

Component	Frequency (%)
1. Self-monitoring: weight-tracking	106 (59.2)
2. Goal setting	99 (55.3)
3. Self-monitoring: body mass index calculator	86 (48.0)
4. Feedback on goal progress	77 (43.0)
5. Planning content and scheduling of diet and activity	48 (26.8)
6. Prescriptive help: menus	48 (26.8)
7. Information: diet	44 (24.6)
8. Prescriptive help: physical activity	43 (24.0)
9. Calorie calculator	43 (24.0)
10. Reminder setting	37 (20.7)
11. Self-monitoring: physical activity tracking	35 (19.6)
12. Motivation	31 (17.3)
13. Self-monitoring: body measurements tracking	27 (15.1)
14. Behavioral strategies: diet	27 (15.1)
15. Self-monitoring: diet tracking	26 (14.5)
16. Self-monitoring: body fat calculator	26 (14.5)
17. Support: motivational	25 (14.0)
18. Self-monitoring: body fat tracking	23 (12.8)
19. Behavioral strategies: physical activity	19 (10.6)
20. Information: physical activity	16 (8.9)
21. Reward	14 (7.8)
22. Weight management aids	7 (3.9)
23. Support: buddying	6 (3.4)
24. Support: professional	3 (1.7)
25. Impulse management	3 (1.7)
26. Reframing	2 (1.1)
27. Self-experimentation	1 (0.6)

**Table 3:** Pearson correlations assessing the association of the complexity of weight loss apps with the three popularity indicators: rating, number of reviews, and number of downloads.

Popularity indicators and complexity		Rating	Reviews	Downloads	Count components
**Rating**					
	Coefficient	1			
	*P*				
	N	170			
**Reviews**					
	Coefficient	.14	1		
	*P*	.08			
	N	169	178		
**Downloads**					
	Coefficient	.19	.33	1	
	*P*	.01	<.001		
	N	170	178	179	
**Count components**					
	Coefficient	.25	.28	.48	1
	*P*	.001	<.001	<.001	
	N	170	178	179	179

### Review Analysis Weight-Tracking Apps

#### Descriptive Analysis

After conducting the second screening, 15 mobile apps remained in the sample (Table 4). The apps’ ratings ranged from 3.6 to 4.6 (mean=4.21) on a scale from 1 (low) to 5 (high satisfaction). The number of reviews lay between 1196 and 121,871 (mean=22084.27), and the number of downloads ranged accordingly from 100,000-500,000 to 10,000,000-50,000,000. From these 15 mobile apps, 569 reviews were sampled for qualitative analysis.

#### Framework Analysis

In the framework analysis, we assessed the frequency and valence of comments addressing specific components of an app. The first most positively commented on group of components was related to weight-tracking and (graphical) feedback (components 1-6 in Table 5). The second most positively commented on group of components was related to reliable data storage (components 7-10 in Table 5).

##### Detailed (Graphical) Feedback

Users frequently highlighted the importance of receiving graphical and nongraphical feedback on their weight loss journey. They appreciated seeing graphs of their weight over time, especially when additional visual cues such as a trend line were given. Comments included:

I like how it graphs out your weight loss to show your progress. It’s very motivating to see how far you have come from where you started.

The trend line is a neat feature to forecast when I’ll reach my goal weight.

Some mobile apps had a component that predicted the date by which the user would reach his or her goal. This function was remarked positively by several users:

I like the way it predicts the date for you to reach your goal and the timeline showing your percentage of progress. The graphs are great too! Very motivating.

Generally, users stated that the feedback provided them with motivation to keep going. Where weight loss success was lacking, users appreciated neutral feedback:

I love the cheering when you lose a bit of weight. That’s very sweet and makes you feel good.

even when you are going through a bad spell it seems non-judgy.

##### Reliable Storage of Data

Users emphasized the importance of reliable storage of their data that was often collected over a long period of time. When the data was lost through a malfunctioning update or a glitch, it caused frustration in the user:

New update deleted all my data. Horrible. What’s the point of making an account if your data doesn’t get stored? I’m so mad!

Back up doesn’t work! I’d been backing up my data weekly to my SD card. Just got my new phone yesterday, went to import database and it says “database empty”—3 years of data lost! Fantastic!

**Table 4:** Basic information about the 15 weight-tracking apps.

App name	Developer	Rating	Reviews	Version	Downloads
Weight Loss Tracker, BMI	Easy Creation	4.6	1457	1.2.11	50,000-100,000
Weight Log & BMI Calculator	aktiWir	4.5	31,455	1.44	1,000,000-5,000,000
Weight Loss Tracker-RecStyle	Recruit Holdings Co., Ltd.	4.4	17,067	3.1.8	1,000,000-5,000,000
Monitor Your Weight	Husain Al-Bustan	4.4	91,617	4.9.2	5,000,000-10,000,000
Libra-Weight Manager	Daniel Cachapa	4.4	19,623	3.3.3	1,000,000-5,000,000
BMI and Weight Tracker	despDev	4.4	16,559	3.4.4	1,000,000-5,000,000
BMI-Weight Tracker	Peytu	4.4	3007	1.97	100,000-500,000
My Weight Tracker, BMI	b3c.apps	4.2	5276	3.4	500,000-1,000,000
Weight Tracker	Pig Dog Bay	4.2	1196	1.18.05	100,000-500,000
Simple Weight Recorder	Beyonj	4.2	3551	-	1000000-5000000
Weight Loss Tracker	Appovo	4.1	1537	1.0.9.8	100,000-500,000
BMI Calculator & Weight Loss	Appovo	4.0	121,871	4.2.4	10,000,000-50,000,000
BMI-Weight Tracker	Monirapps	4.0	1560	3.3	100,000-500,000
Weight loss and fitness app	cryofy.com	3.8	13,464	1.3.6	1,000,000-5,000,000
A+ Weight Manager Lite	Mobileking, Inc.	3.6	2024	1.6	500,000-1,000,000

**Table 5:** The top 10 most positively commented on components. Improvement comments refer to comments where users suggested or demanded the improvement of a specific aspect of the app.

Component	Frequency of positive comments	Frequency of improvement comments
1. Weight-tracking	70	4
2. Graph feedback	51	22
3. Target line graph	48	0
4. Feedback goal	35	1
5. Prediction target date	34	0
6. Trend line graph	27	2
7. Google Drive	25	3
8. Sync Google Fit	22	12
9. Backup cloud server	19	1
10. Import data	18	9

**Table 6:** The most prominent themes identified through thematic coding.

Themes and nodes		N^a^
**Simplicity or ease of use**		
	Easy to use	133
	Simple	73
	Right amount of complexity	14
**Smooth functioning**		
	Technical issues	66
	Smooth working	26
**Long-term app usage**		
	Long-term use	54

^a^N: the frequency of comments addressing the respective node.

Following the loss of data, many users stated that they would discontinue their usage of the app and switch to a different one:

Lost all of my tracking history upon update. Even update on 11/16/16 did not correct the issue. All tracking ID just gone. Time to uninstall.

I was using this track [sic] my weight loss over a year. Updated and lost everything. Going to try a different app now.

Users appreciated mobile apps that allowed them to protect their weight data with a password:

...love that can back up data and put password on it.

A pin code for privacy on the app would be great.

#### Thematic Coding

In addition to the comments that directly addressed specific components of the app, we also conducted inductive thematic coding of user comments. The three most frequently identified themes were simplicity and ease of use, smooth functioning, and long-term app usage (Table 6).

##### Simplicity and Ease of Use

Users of weight-tracking apps expressed a preference for simple apps, equipped with only a few key features. These key features were mostly weight logging and graphing. Elaborate additional components were perceived to add unnecessary complexity to the app:

Just what I needed and nothing more to clutter the screen. I was looking for an app to log my weight and graph it. This got it right.

I love this app because it is simple and just does exactly what I want instead of a bunch of extra stuff that I’m not going to use and is just going to be in the way.

Reviewers commonly expressed a preference for app designs that were intuitive and easy to use, allowing for efficient app usage:

I can log data in seconds without navigating through needless steps.

##### Smooth Functioning and Technical Issues

The smooth running of the app emerged as a main criterion for user satisfaction and was critical to user retention:

Been using for a few years, it runs smooth and does what it says it will, which is tough to find sometimes.

Most reports of technical issues reflected problems with an update of the app, suggesting developers were not always able to meet the needs and demands of their users when releasing a new version:

As seems to be the norm these days, every time a good app is “updated” it gets ruined. Updated on 11/11 and visibility is terrible and takes ages to display main screen.

Nov update has ruined this app. Won’t use anymore.

...lost all my data after upgrade, behaves really odd, locks up! used to love it...

##### Long-Term Usage

In 54 of the 569 reviews, people commented on their intention to use a weight-tracking app on a long-term basis:

Been using it for years and always been reliable.

Has been my Go-To weight tracking app for several years now, can’t fault it :)

Once users found the app that best fits their needs, they were willing to remain loyal to the app and even downloaded it onto new devices:

Every time I change my phone, this app is a must to be downloaded.

##### Additional Themes

There were several other less prominent themes. Users appreciated it when developers acted mindfully with memory space. Either keeping the size of the app to a minimum or storing the app onto the secure digital card automatically received positive attention by the users:

Does exactly what you need without it being a bloated app that gobbles up much needed memory.

As someone who gets annoyed with my tiny phone memory filling up, the fact that this not only can be stored on a SD card but actually automatically saves to it is brilliant.

Similarly, users appreciated the mindful deployment of advertisements. Inappropriate advertising (such as for a fast food chain) or impairment of app functionality through advertisements were criticized:

What’s up with the Duncan donut add every time I open.

Too many ads. Covers the page so you can’t enter stats.

Inaccuracy of in-app calculators (such as BMI and body fat) was perceived as frustrating. Users criticized the inaccuracy harshly and expressed disbelief:

I’m sevearly [sic] underweight and on and off I’ve been hospitalized for it so when I calculated my weight and height I was very shocked to see that apparently I’m overweight and my 139 pound friend is obese class 1.

Providing options to customize the appearance of the app and its functionality was well perceived. Users liked personalizing aspects such as background color, font style, or the amount of measurements to be tracked:

I wish you could customize the widget colors, font size, etc.

...love it if I could personalise the colours in the app.

Finally, we found that when users were satisfied with an app, they stated their willingness to spend money on premium features or blocking of advertisements:

Happily paid the few $ for the pro version after trying the free one for a couple months.

...but I haaaaate the update that has now put ads in the app. I totally understand the developer needing to make money, put I’d rather pay for a premium version to get rid of the ads.

## Discussion

### Principal Findings

Our results provide insights into the preferences of app users. In correlational analyses, including all weight loss apps, we found that more complex apps had higher ratings, more reviews, and more downloads, suggesting higher popularity. In our review analysis of weight-tracking apps, we found that components related to feedback on the weight loss journey were of high importance. Receiving positive feedback and visualizing weight loss success provided the user with positive reinforcement and thus increased motivation. Furthermore, users emphasized the importance of reliable data storage as they intended to use the app on a long-term basis. Users appreciated mobile apps that were specialized, with few additional components unrelated to weight-tracking and that were intuitive and easy to use. Users emphasized that the smooth functioning of the app is of immense importance. When technical issues arose, users appeared likely to discontinue using the app.

### What Weight Loss Apps Offer

In our component analysis of general weight loss apps, we found that more than half had components related to weight-tracking and goal setting. Another 43.0% (77/179) of the apps provided users with feedback on their goal progress. This finding resembles previous content analyses of weight loss apps. For instance, Bardus and colleagues assessed behavior change techniques (BCTs) of weight management apps in 2015 and found that the most prominent ones were based on self-monitoring, goal setting, and feedback [16]. Similarly, Pagoto’s review found that goal setting and self-monitoring were the most prominent behavioral strategies in their sample of weight loss apps in 2012 [8]. This suggests that the content of weight loss apps is quite stable, as the same components remain prominent over time. Importantly, these three component types are well-studied BCTs that are known for their effectiveness in fostering a healthier lifestyle [26].

Fewer than 20% of the 179 weight loss apps in our sample offered social support features. Social support can contribute to weight loss success, as has been shown in randomized studies comparing individual- with group-based approaches [27,28]. It is therefore surprising that only few apps made use of the technological opportunities to connect users with other weight loss seekers around the world. The lack of social support components has already been criticized by Breton and colleagues in 2011 after they had conducted an app market review of weight control apps [2]. However, the implementation of social support features in mobile apps is costly to build and maintain. It is therefore conceivable that only the most economically valuable (ie, popular) mobile apps such as *Lose It!* or *MyFitnessPal,* are able to offer these features.

### What Users Find Important

In line with our results of the content analysis of weight loss app components, the most positively commented on components in the review analysis were also related to self-monitoring and feedback on goal progress. Our finding that users perceived feedback on and visualization of their weight loss progress as useful and motivating is consistent with the findings of other studies [29,30]. Burke and colleagues showed that self-monitoring with feedback helps participants achieve more weight loss than self-monitoring alone [23]. They suggest that feedback provides guidance and accountability to the user, regardless of the valence of this feedback. Although users in our sample did not state they felt accountable to their device, they did state that the feedback kept them on track with their weight loss goal. Users liked to receive as many details about their progress as possible to gain the most reward out of a satisfactory outcome. In a qualitative study by Dennison and colleagues, participants expressed their concern that they might find corrective feedback and lack of success particularly daunting and deterring in weight loss apps [30]. The results of this review do not support these concerns; lack of success was mostly responded to with neutral feedback, and users did not feel negatively judged for their lack of progress.

Beyond the weight management components, users expected high levels of technical reliability and secure data storage. This is critical because users often intend to use a weight-tracking app on a long-term basis, meaning that these apps often cover years of data. This data is often of emotional value, as it provides motivation to the user and visualizes previous successes and hard work. Losing data that has been collected for several years is perceived as immensely frustrating and can lead to a loss of trust in the app.

In addition, reliable data storage is also important from a user retention perspective: there are many weight-tracking apps available on the app store market, so initially users can easily switch from one to another, and they often try out several at a time [30]. Switching barriers are only created once a user starts collecting data in one app over a considerable amount of time, as this data is usually not transferable between mobile apps. A loss of data eradicates this additional value of the app, making it easier for users to switch to another app.

Reviewers highlighted the importance of an intuitive and simple app design, which is consistent with findings in the literature [29]. A qualitative study by Dennison and colleagues showed that users do not have a lot of patience in dealing with mobile apps and quickly discard them when usage is perceived as complicated [30]. Hence, a simple and straight-forward design is critical.

With regards to simplicity, we found conflicting results for the weight loss compared with the weight-tracking apps. Whereas the correlational analysis showed that complex weight loss apps are more popular than simple ones, the review analysis of weight-tracking apps indicated that users prefer simple and basic apps. One possible explanation for the finding that more complex weight loss apps had higher popularity ratings could be that these complex apps met the needs of more users. People seeking to download a weight loss app for the first time might settle for a more general and comprehensive app than a specific one in order to be able to test several strategies for weight loss. Once they have found the right features for them, they might download an app that is more specific to their needs. This could explain our finding that for weight-tracking apps, less complexity and fewer components were preferred by the users. Further research is needed to examine this.

A substantial amount of reviews contained complaints about the malfunctioning aspects of a given app. Most of these complaints were related to dissatisfaction with an update of the app. The topics of the reviews ranged from the emergence of technical issues and the lack of a previously existing feature to disliking of a new user interface design. This suggests that updated versions were premature and not sufficiently tested on users before they were made available to the public. Similarly, in a study analyzing complaints in user reviews from the top 20 apps on the iOS app store, Khalid and colleagues also found that a substantial number of comments originated from issues with an update [31]. They concluded that developers should engage in more rigorous testing and work more closely with users before introducing new versions of an app. They warned that user frustration with an update can lead to bad ratings of an otherwise good app. On the other hand, we found that updates can also lead to positive outcomes. In our review, there were several cases in which reviewers commented on positive changes to issues they complained about or edited their negative ratings to more positive ones when problems were resolved. Similarly, a recent analysis showed that nearly 50% of user suggestions are implemented in app updates and that this consideration of user opinions is rewarded with higher app ratings [32]. Hence, involving users in the development of an app is highly recommended.

Another approach to enhancing user satisfaction can be to allow for personalization of the app. In our review, users appreciated the opportunity to adapt the app to their needs and likes, for instance, by allowing for the choice of a theme or design. Similarly, Dennison and colleagues found in their study that users liked to be able to make personalized settings such as deciding when to receive reminders to weigh [30]. Generally, allowing for personalization enables the developer to cater more needs and preferences and therefore attract more users.

### Strengths and Limitations

A major strength of this app market review is that we analyzed the voices of a large and diverse sample of people. The content of app reviews is influenced by the needs and expectations of the user, as well as the context of usage and can therefore vary considerably. Asking only a small group of people, such as Dennison and colleagues’ focus group, can restrict the variety and representativeness of the data. With our large sample, we were able to see patterns and analyze which concerns are shared by most users and which ones are more specific. This allows us to make more reliable conclusions about what users like and dislike in mHealth apps. Importantly, to the best of our knowledge, we are the first to use this method of qualitatively analyzing user reviews of weight loss apps. We found this process to be very fruitful for gaining insights into user experience and opinion.

One limitation of our review is that not all of the recommendations we conclude from our results will be generalizable to other kinds of apps. That is, it is conceivable that the most important aspects that users are concerned about differ between app categories. For instance, the importance of feedback on a goal is most probably specific to self-monitoring apps and will not be of imminent importance for, for example, gaming apps. However, concerns about reliable data storage, ease of use, and app updates most probably pertain to other app categories.

It is also possible that people who write user reviews on the app store are not necessarily representative of the broader population of app users. Nevertheless, our approach to reviewing user experience allowed us to capture feedback from a large number of users and hence, provided us with a more diverse sample than would have been achieved through commonly used methods such as qualitative interviews or focus groups.

Another limitation of this review is that we only considered mobile apps from the Google Play Store. This decision was due to a lack of similar indexing methods and technological issues with the download of user reviews from the iOS store. However, a recent analysis of the mHealth marketplace has concluded that 75% of today’s health and fitness app developers produce mobile apps for both the Android and iOS market [1]. The two markets therefore overlap substantially, and it is questionable whether we would have found any meaningful differences between the two stores. By searching the app store directly rather than through a search engine, our results are limited by the fact that the Google Play Store only displays 249 mobile apps per search term. Our approach was guided by the intention to analyze those mobile apps that users of the Google Play Store can find. However, several more apps would have been found when using a search engine.

Finally, our results are limited in that this review focused on popularity but not on the effectiveness of discussed mobile apps. However, reviews assessing the evidence-base and potential effectiveness of mHealth apps can be found elsewhere in the literature, where the occurrence of BCTs has been used to evaluate their quality [5]. Moreover, when comparing our results with the literature, we find that effectiveness and popularity partly overlap, as well-accepted and effective BCTs such as self-monitoring and feedback [13,33] are particularly liked by users.

### Conclusions

This review extends the literature by highlighting which facets of weight loss apps are especially important to users and should be considered when developing an app. In particular, findings from this review suggest several aspects of app design that developers of self-monitoring apps should consider. First, developers should focus on providing appropriate feedback on goal progress, as users appeared to respond favorably to detailed feedback on weight loss success and appreciated it when mobile apps remained neutral when success was lacking. Second, we recommend that developers make sure that data is stored reliably to enable people to use the app on a long-term basis. Third, developers should keep in mind who the chosen target audience is. A simple and basic app version may be sufficient when speaking to a specifically targeted audience. However, more comprehensive weight loss apps may attract more people, as they speak to anyone who is generally looking into weight loss support. Fourth, we recommend that developers incorporate users’ opinion in all design and development stages to ensure that the app fulfils users’ expectations. Furthermore, it is important to perform rigorous testing on an app before making it available to the public, as smooth functioning is critical to user satisfaction and long-term usage. Overall, we believe that our novel methodology and large sample size strengthen our conclusions. We recommend that future app market reviews assess user experience in addition to the evaluation of app components.

## Appendix

Multimedia Appendix 1 Details of all 179 weight loss apps.

Multimedia Appendix 2 Features of the second component coding.

## Abbreviations

BCT: behavior change technique
BMI: body mass index
mHealth: mobile health
OxFAB: Oxford Food and Activity Behaviors
PABAK: prevalence-adjusted and bias-adjusted kappa
SD: standard deviation
